# Pathology of Phlegmasia Dolens

**Published:** 1844-01-13

**Authors:** 


### RECORD OF MEDICAL SCIENCE.

### PATHOLOGY OF PHLEGMASIA DOLENS.

*Discussion at the Royal Academy—Remarks.*

At a recent *seance* of this learned body, M. Capuron read an elaborate report on a memoir by M. Droussard on the disease known by the name of Phlegmasia Alba Dolens, and in which the author has attempted to prove that it is generally, if not always, connected with an inflammation of the crural and other veins of the affected limb. The question, it will be seen, gave rise to much discussion and difference of opinion; some assenting to this view of the subject, and others expressing their decided dissent. The result of the whole seems to be, that there are different forms of the disease, and that these are most probably owing to the operation of different causes.

M. Breschet objected to the opinion, expressed in the report just read, that *phlegmasia alba dolens* is in all cases the result of an inflammation of the veins in the affected parts. According to his experience, the lymphatic vessels are usually as much implicated in the morbid process as the veins. The phenomena of genuine phlegmasia are certainly not the same as those of ordinary phlebitis; and this, among other reasons, he considered a strong argument against the opinion of M. Capuron.

M. Capuron, in reply, said that he had by no means denied that the lymphatic vessels may be inflamed in this disease; but only that they are not primarily and necessarily so.

M. Blandin:—"It has been long imagined that phlegmasia alba is a disease peculiar to women.—This is not strictly correct; for, although of much more frequent occurrence in females, it is certainly not peculiar to them. When the disease was first recognised and described, it seems to have been regarded as a mere form of œdema; but, in proportion as its phenomena and causes were more attentively studied, it was found that in some cases the veins, in others the lymphatic vessels, and in others still the nerves were in a more or less decided state of inflammation at the time:—this M. Dance shewed in his interesting memoir on the subject. Subsequently, however, it has been discovered that the inflammation of the veins and that of the nerves are not a necessary, but only of an occasional, coincidence in phlegmasia; and that the only essential pathological character of the disease is an inflammation of the absorbent vessels of the limb. We must admit, however, that the inflammation of the veins sometimes precedes that of the absorbents, and we are therefore obliged to dissent from the opinion of our colleague, M. Breschet, who seems to regard the last-named vessels as invariably the seat of the disease in question."

M. Velpeau:—"Twenty years ago, when I published my first observations on this disease, it was almost universally maintained that the pathological cause was an inflammation of the lymphatic vessels of the limb. In consequence of several post-mortem examinations, I felt convinced that the veins were often more or less affected, and I expressed this opinion publicly. About the same time, Dr. Davis came to nearly the same conclusions in England. I have had very many opportunities, since that time, of studying the diseases of the venous and lymphatic systems, and have treated not a few cases of phlegmasia alba, and I now feel assured of the truth of the following two propositions: *first*, that the opinion of M. Dance and others, as to the nerves being in some cases inflamed, is utterly erroneous; and, *secondly*, that the disease is primarily seated in the lymphatic vessels of the thigh; that a phlebitis is not necessarily existent; and, when it is, that it is only of consecutive or secondary development. But while I say this, I must also distinctly state that, in all post-mortem examinations of the affected parts, both sets of vessels have been found more or less seriously diseased. The phenomena of the disease in its first stage tend much to show that it is rather an *angeioleucitis* than a *phlebitis* that we have to deal with.—There is then a general engorgement of the limb, with patches here and there of diffused redness, and irregular uneven nuclei or nodules of tumefaction, which are not necessarily found along the *trajet* of the veins, as in genuine phlebitis. The constitutional symptoms, too, in proper phlegmasia dolens, are certainly not the same as in phlebitis; and it is only when both sets of vessels happen to be simultaneously implicated that we ever meet with the symptoms which denote the occurrence of purulent resorption and infection. In conclusion, I will briefly repeat my opinion that genuine phlegmasia alba dolens is attributable to an angeioleucitis or inflammation of the lymphatic vessels, accompanied with an inflammation of the adjacent cellular tissue, and occasionally also with inflammation of one or more of the veins of the limb."

M. Capuron here reminded the Academy that the opinion now expressed by the preceding speaker differs in almost every essential particular from that which he expressed not long ago, and pointed out the striking contradiction between M. Velpeau's present sentiments and those which he has published in his writings.

M. Cloquet:—"I agree with M. Blandin in the sentiment which he has expressed in his remarks, that phlegmasia alba is not necessarily or invariably met with in the female sex; as cases of it have unquestionably occurred, in my own practice, among youths and men. I have experienced no little difficulty in forming any exact or definite opinion as to the ætiology of this disease. If I were to trust exclusively to the results of the post-mortem examinations which I have made, I should indeed be utterly perplexed what to say; but, by having carefully watched the progress of many cases from their first stage, I have been enabled to form clearer and more accurate notions, as I think, on the subject under consideration. The result of my observation is that, as a general remark, the cellular tissue is primarily the seat of the œdematous swelling; that an inflammatory engorgement, accompanied with effusion, takes place at first; and that it is only consecutively that either the lymphatic vessels or the veins become affected. In my opinion we may regard phlegmasia alba as a specific exhalant inflammation of the cellular tissue, with or without an accompanying inflammation of the veins or lymphatic vessels of the part."

M. Moreau (the eminent obstetrical physician) took nearly the same view of the ætiology of phlegmasia alba as M. Cloquet. In his opinion, it is a specific disease which should not be confounded with an inflammation either of the absorbents or of the veins." "Phlebitis is always a serious, and often a most dangerous, disease; phlegmasia alba, when uncomplicated with other lesion, is comparatively mild and innocuous. In proof that there is something special about the disease, we have only to bear in mind the circumstances which usually give rise to it. Is it not remarkable that often it does not occur for fifteen, eighteen, or twenty days after delivery, when the usual exciting causes of suppuration have entirely passed away? Does not this circumstance alone suffice to show that the disease depends most frequently on the neglect of certain precautions on the part of women who have been recently delivered? According to my own experience, it is generally owing to checked perspiration under such circumstances. At first there seems to be nothing but an indolent swelling, a simple engorgement; this, if not relieved, is apt to be followed by inflammation of the absorbents, and in some cases of the veins also. It is only when the last named lesion is present, that there is any cause for alarm as to the issue of the case. I should define the disease in its primary stage to be 'une veritable inflammation exhalante du tissu cellulaire.'"

M. Berard:—"I have listened with a great deal of pleasure to the remarks which have just been made by MM. Cloquet and Moreau; for I must frankly confess that I have never very clearly understood what part the alleged inflammation of the absorbents and veins has been supposed to act in the history of phlegmasia dolens. When the superficial absorbents of an extremity are really inflamed, the phenomena are usually sufficiently obvious to prevent any mistake in the diagnosis; and certainly these phenomena are not those generally exhibited by the disease in question.

With crural phlebitis, it might be more easy to reconcile the symptoms; for M. Bouillaud has shown that an adhesive inflammation of a vein is apt to be followed by œdematous swelling of the part and its neighbourhood, in consequence of the obstruction to the free return of the blood. But, notwithstanding this plausible explanation, I must confess that I am much more inclined to adopt the pathological views of MM. Cloquet and Moreau. There is a fact which, although not bearing directly on the subject under consideration, may not be undeserving of notice here; I allude to the tendency which exists in puerperal women to the formation of diffused abscesses."

M. Andral:—"The expression, phlegmasia alba dolens, is a very unfortunate one, and has tended not a little to keep up the obscurity that hangs over the subject: it is a complex term that embraces a variety of morbid alterations dissimilar from each other, and thus tends to perpetuate confusion. I agree with those gentlemen who have asserted that the disease is not confined (as alleged by M. Rochoux) to the female sex. I have seen unquestionable cases of it among men; but in them, as well as in women, always associated with a lesion of some of the pelvic viscera. I feel satisfied of the truth of this position, that wherever there is a phlegmasia dolens, we may at once suspect that there is something wrong within the pelvis. The 'point de depart' of the disease is an obstruction of the chief vein of the limb; this is the cause of the œdema.

"In every case which I have examined by dissection, I have found almost uniformly a correspondence between the lesion of the veins and some inflammatory process within the pelvis—the diagnosis of which, it must be confessed, is often exceedingly difficult during life.

"But I do not mean to assert that this complex disease is always, although certainly generally, dependent upon a mere obliterative phlebitis. Besides the lesion of the veins, there may be a lesion of the absorbent vessels, and also of the cellular tissue. In women the disease is generally a result of delivery; in men, either of some morbid affection within the pelvis, or of an injury of the lower parts of the limb."

M. Gerardin expressed his surprise that none of the preceding speakers had alluded to the connexion that certainly exists between the occurrence of the disease, at least in puerperal women, and the state of the mammary secretion. It is acknowledged by all writers, that it is more frequent in those who do not suckle their children than in those who do; and the sudden suppression of the milk has often been known to be quickly followed by an attack of phlegmasia dolens, as well as by sudden effusions into the cavity of the chest, abdomen, or of some of the joints.

Now the diseased action, in both sets of cases, is nearly the same; in the one, the effusion takes place into a cavity, while in the other, it is into the subcutaneous cellular tissue. It is sometimes surprising to see with what rapidity such effusions are induced; their gradual increase may be watched from one hour to another. It is usually asserted that phlegmasia alba is of much more frequent occurrence in the lower than in the upper extremities. Now the very reverse seems to me to be the case. (We do not remember having ever met with any statement to this effect before.) It has also been said that the disease is seldom attended with any danger; but such an opinion must be received with due caution, as a sudden effusion may take place into one of the internal cavities, or into the sheaths of the blood-vessels, and prove fatal either rapidly, or by a slow wasting process.—*Gazette Medicale.*

*Remarks.*—The attentive reader cannot fail to have remarked the great and more than usual discrepancy of opinion among a set of eminent men, the members of one of the first medical societies of the age, as to the nature of a disease which is not of very unfrequent occurrence. We are told by one learned academician that the veins are the parts most affected; by another, that the lymphatic vessels are usually the seat of the morbid action; by a third, that both sets of vessels, and the nerves of the limb also, are always more or less inflamed; and by a fourth, that the disease is quite independent of all these parts, and is truly and essentially one of the cellular tissue. May we not, *á priori*, suspect from this very discordance of opinion, that the truth really lies, not in one nor in another of these doctrines, but in the *ensemble* of all put together; and that the disease in question does not uniformly or invariably commence in one part alone? Perhaps it might be said with more accuracy, that, under the name of phlegmasia dolens, different diseases, more or less dissimilar from each other, are grouped together as if they were all cognate affections.

For our own part, we are certainly inclined to think so, and we cannot express our surprise that so accurate and cautious a pathologist as M. Andral is, should regard an œdematous swelling of the thigh, supervening upon an injury of the foot or leg, as analogous with genuine phlegmasia dolens.

In studying this, as well as every other puerperal affection, we should never lose sight of the peculiar state of the constitution in which the morbid action is apt to occur; viz. within from one to three weeks after delivery.

The loss of blood, the exhaustion of nervous energy, the excitable state of mind, the secretion of the milk, and the systematic disturbance that so often accompanies it, the distension of the cellular tissue,

&c., are so many circumstances which must render the puerperal state different from each other, even among females themselves, and which stamp with a peculiar character the diseases that are then apt to occur. No experienced physician will ever lose sight of this most important consideration; and if it had been less neglected than it has been too often during the present century, we should have less cause to lament the not very creditable controversies on puerperal fever, and the melancholy loss of life that has (on some occasions at least) been the consequence.

We have little doubt, in our own minds, that in a large majority, if not in all, cases of genuine phlegmasia dolens, there is some affection of the uterus, cotemporaneous with the outward and visible one of the thigh. We generally find that there is a greater or less degree of tenderness in the corresponding iliac region, or even the pubes, at the commencement of the attack, and the lochial secretion is always more or less disturbed. That this uterine affection is of an inflammatory nature is more than probable; but then, be it remembered, the character of the inflammation has something unusual and special; it has, if we may so speak, a peculiar idiosyncrasy; and it is this very peculiar idiosyncrasy that makes all the difference—and a most important one it is—between such an œdema of the limb as may supervene upon an injury of the foot, and the proper phlegmasia dolens of puerperal women.

Now, although we confidently deny that phlegmasia alba is either a phlebitis, or an angeioleucitis, or a neuritis, or all three together, we are ready to admit that each or all of these affections may exist simultaneously with it. Such complicated cases are, as a matter of course, more unfavourable than the others, and they will necessarily require certain modifications in the line of treatment to be pursued.

It is certainly much easier to say what the disease is not, than what it really is; and we must frankly admit that its pathology is (in spite of the researches of numerous observers) still very far from being satisfactorily made out.

With respect to treatment, the only safe plan is minutely to examine all the phenomena of each case by itself, and, independently of any preconceived hypothesis as to its nature, to watch its progress from day to day, and to adapt our remedies to the symptoms as they develop themselves, always paying the greatest attention to the state of the uterus, and keeping in mind the peculiarities of the female system in the puerperal state. — *Medico-Chirurgical Review.*

